# IL-21–Deficient T Follicular Helper Cells Support B Cell Responses Through IL-27 in Patients With Chronic Hepatitis B

**DOI:** 10.3389/fimmu.2020.599648

**Published:** 2021-01-28

**Authors:** Arshi Khanam, Natarajan Ayithan, Lydia Tang, Bhawna Poonia, Shyam Kottilil

**Affiliations:** Division of Clinical Care and Research, Institute of Human Virology, University of Maryland School of Medicine, Baltimore, MD, United States

**Keywords:** chronic hepatitis B, T_FH_ cells, interleukin-27, B cells, interleukin-21

## Abstract

Chronic Hepatitis B (CHB) affects over 350 million people worldwide. Current treatment does result in reduced complications; however, a cure (development of antibodies to the S antigen) is not achieved, requiring life-long therapy. Humoral responses contribute to viral elimination by secreting neutralizing antibodies; though, effective induction of humoral immunity require CD4T cell differentiation into T follicular helper (T_FH_) cells that support B cell response through interleukin-21 (IL-21). In CHB, mechanism of T_FH_-B interactions is seldom described. During CHB, T_FH_ cells are defective in producing IL-21 in response to hepatitis B surface antigen (HBsAg). However, regardless of low IL-21, T_FH_ cells efficiently support B cell responses by producing interleukin-27 (IL-27), which directs the formation of plasmablasts and plasma cells from memory and naïve B cells by enhancing B lymphocyte-induced maturation protein-1. IL-27 not only improved total antibody production but HBsAg-specific IgG and IgM secretion that are essential for viral clearance. Importantly, IL-27+T_FH_ cells were significantly associated with HBV DNA reduction. Therefore, these findings imply a novel mechanism of T_FH_ mediated B cell help in CHB and suggest that IL-27 effectively compensate the function of IL-21 by supporting T_FH_-B cell function, required for protective antibody response and may contribute to viral clearance by providing potential target for achieving a functional cure.

## Introduction

Chronic hepatitis B (CHB) infection represents one of the most common viral diseases affecting approximately 350 million people worldwide (1, 2). The magnitude and character of the T-cell response and development of hepatitis B surface antigen (HBsAg)-specific antibody, primarily dictate the outcome of hepatitis B virus (HBV) infection. HBsAg-specific antibody production is the major clinical correlate of resolution of infection. However, during CHB, T cell exhaustion and inadequate humoral response facilitate virus replication (3) and progression towards fibrosis, cirrhosis and hepatocellular carcinoma (HCC).

CD4+CXCR5+ T follicular helper (T_FH_) cells are primarily associated with B cell response and are essential for the development of germinal centers (GCs) from which high-affinity memory B and long-lived plasma cells are generated, which are crucial for the development of protective antibody response (4, 5). This phenomenon requires T_FH_ cell migration to the lymphoid organ. Since it is not feasible to get these organs from humans and analyze tissue resident cell populations, considerable efforts have been made to study circulating T_FH_ cells, which could imitate the environment of lymphoid tissue. In fact, circulating CXCR5^+^PD-1^+^CXCR3^-^T_FH_ cells induces antibody response which correlates with germinal center T_FH_ cells (6).

Persistent viral infection encourages the differentiation of CD4+T cells into different lineages including T_FH_ cells (7). Several studies reported that T_FH_ mediated B cell proliferation and maturation is regulated by Interleukin-21 (IL-21), a hallmark T_FH_ cell cytokine that acts through its cognate receptor present on B cells (8, 9). IL-21 promotes the differentiation, proliferation and class switching in B cells (10) (4, 11–13), and is critical for the regulation of germinal center and humoral immunity (14). However, a study reported that during chronic hepatitis C virus (HCV) infection, T_FH_ cells produce extremely low IL-21. This, however did not restrict B cell responses, giving rise to plasmablasts and IgG production in quantities similar to healthy controls in whom large quantities of IL-21 were seen (11), which suggest that T_FH_ cell mediated B cell help is complex and multiple factors not restricted to IL-21, co-stimulatory molecule CD40L (15), inducible co-stimulator (ICOS) (16) nor co-inhibitory molecule programmed death-1 (PD-1) (17) are involved. Development of HBsAg-specific humoral response is the widely accepted clinical protective correlate in HBV infection. Hence, in the present study, we hypothesize that T_FH_ cells are capable of supporting B cell responses using alternate mechanisms that compensate for IL-21 function and facilitate B cell help. To the best of our knowledge, our study is the first to demonstrate that T_FH_ cells produce interleukin-27 (IL-27) which supports T_FH-_B cell interactions and helps in B cell functions. Although the role of IL-27 has been reported in the development of T_FH_ cells, it has been shown that IL-27 enhances the expression of T_FH_ cells markers and is critical for the function of T_FH_ cells and for normal and pathogenic GC responses (18); however, whether T_FH_ cells themselves produce IL-27 remains completely unknown. Our study revealed that IL-27 produced by T_FH_ cells help in the generation of plasmablasts and plasma cells from both memory and naive B cells by inducing B lymphocyte-induced maturation protein-1 (Blimp-1) expression, essential for protective antibody response. Furthermore, our data demonstrates that IL-27 not only increased total immunoglobulin secretion but also enhanced HBsAg-specific IgG and IGM secretion by B cells, required for viral clearance and associate with HBV DNA reduction.

## Materials and Methods

### Patients

CHB patients were enrolled in a natural history study (Hope NCT02995252) at the Institute of Human Virology, University of Maryland School of Medicine or one of our collaborating clinics. The Hope study protocol (NCT02995252) was approved by institutional review board of the University of Maryland School of Medicine, Baltimore, MD, USA. Written informed consent was received from all subjects. CHB patients were HBsAg and anti-HBc positive, anti-HBs negative, and treatment naive. None of the CHB patients had cirrhosis, co-infection with another viral hepatitis, autoimmune hepatitis or human immunodeficiency virus (HIV) infection.

HBsAg-vaccinated healthy control (HC-Vacc) who had standard schedule of HBV vaccination at 0, 1, and 6 months were taken as controls. All the HC-vacc were positive for anti-HBs antibody. Prior to enrolment, healthy individuals were screened for liver disease and presented no medical history of liver disease or alcohol abuse.

### Samples

For phenotypic and functional profiling of T_FH_ and B cells, 20 ml peripheral blood sample was taken from HC-vacc and CHB by venipuncture and collected in heparin containing tubes. For sorting of T_FH_ and B cells intended for co-culture experiment, 50 ml of peripheral blood was drawn. Plasma was separated after centrifugation of blood samples at 4000 rpm for 5 minutes and stored at -80^0^ until use. Peripheral blood mononuclear cells (PBMCs) were isolated by density gradient centrifugation, frozen in fetal bovine serum containing 10% dimethyl sulfoxide and stored at −140°C in liquid nitrogen.

### Phenotypic Analysis of T_FH_ and B Cells

For immunophenotyping, PBMCs were thawed in complete RPMI1640 medium (10% FBS, 1% glutamine and 1% penicillin and streptomycin) and rested overnight at 37^0^ in CO2 incubator. Cell viability was checked by trypan blue dye exclusion assay which demonstrated >90% viability. To identify the frequency and different subset of T_FH_ cells, PBMCs were surface stained with anti-human CD3 AF700, CD4 PerCP-Cy5.5, CXCR5 BV421, CCR4 BV510, CCR6 BV605, CCR10 APC, CXCR3 FITC, PD-1 PeCy7, ICOS-PE antibodies in 96 well round bottom plate for 30 min at 4° in dark. Cells were washed with 1× PBS at 1300 rpm for 5 minutes. For B cells, PBMCs were first surface stained with CD19 FITC, CD24 BV605, CD27 BV510, CD38 PerCP-Cy5.5, CD138 PeCy7, IgD AF700, IL-27Rα PE and then intracellular staining with BLIMP-1 APC was performed after fixation and permeabilization with eBioscience FOXp3/transcription factor staining buffer set (Cat no. 00-5521-00) according to the manufacturer’s instructions. After 2× PBS wash, paraformaldehyde was added at a conc. of 0.5% and cells were acquired on flow cytometer (BD FACSARIA II). Maximum number of events were collected. Data analysis was done with flowJo v10 software.

### Detection of T_FH_ Cytokines

To examine the function of T_FH_ cells, both HBV antigen specific and global cytokine expressing T_FH_ cells were analyzed. To measure the HBV-specific T_FH_ cell responses, 1 × 10^6^ PBMCs from HC and CHB patients were incubated in complete RPMI1640 medium in 48 well plate and cells were stimulated with PepMix HBV large envelop protein Ultra (HBs) which has a mix of 216 peptides (15mers with 11 aa overlap) derived from large envelop protein of HBV designed to cover the high sequence diversity of HBV (Product code PM-HBV-LEPULTRA, JPT innovative peptide solutions) and PepMix HBV capsid (HBc) protein which has a pool of 44 peptides derived from a peptide scan through capsid protein of HBV at a conc. of 1 μg/ml (Product code PM-HBV-CP, JPT innovative peptide solutions) for 5 days at 37^0^ and 5% CO2 along with CD49d and CD28 co-stimulation (2 μg/ml) (Clone MQ1-17H12, Cat no. 500301, BioLegend). On day 4, cells were restimulated with HBs and HBc peptides for 18 h. For detection of global T_FH_ cell cytokines, PBMCs were cultured in 96 well flat-bottom plate and stimulated with 500 ng/ml of Phorbol 12-myristate 13-acetate (PMA) and 1 μg/ml ionomycin for 18 h at 37^0^ and 5% CO2. After 2 h of incubation, 1 μg/ml protein transport inhibitor (golgi plug containing brefeldin A (Cat no. BDB555029, Fischer Scientific) was added. Following stimulation, cells were washed twice with 1× PBS and stained with live/dead fixable far red dead cell stain (Cat no. L10120, Invitrogen) for 30 minutes and further staining was performed as per above mentioned protocol using anti-human antibodies including CD3 AF700, CD4 PerCP-Cy5.5, CXCR5 BV421, PD-1 BV510, IL-4 PeCy7, IL-17A PE, IL-21 AF647, IL-22 PeCy7, IL-27p28 APC, IL-27EBI3 PE, and IFN-γ BV605 in different panels. Details of the antibodies used in this study has been given in **Table 1**. Cytokine producing cells were analysed in CD4+CXCR5+ T_FH_ cell population as well as CD4+CXCR5+PD1^+^ and CD4+CXCR5+PD1^-^ T_FH_ cells. Moreover, to investigate the functional status of PD1^+^ and PD1^-^ T_FH_ cells, expression of CD69, an activation marker, was analysed on both PD1^+^ and PD1^-^ T_FH_ cells after stimulation with HBs, HBc peptides and PMA/ionomycin.

**Table 1:** Details of the antibodies used in this study.

Antibody	Fluorochrome	Clone	Catalog no.	Company
CD3	AF700	UCHT1	300324	BioLegend
CD3	APC-Cy7	HIT3a	300318	BioLegend
CD4	PerCP-Cy5.5	A161A1	357414	BioLegend
CXCR5	BV421	J252D4	356920	BioLegend
CXCR3	FITC	G025H7	353704	BioLegend
CCR4	BV510	L291H4	359416	BioLegend
CCR6	BV605	G034E3	353419	BioLegend
CCR10	APC	6588-5	341505	BioLegend
PD-1	PeCy7	EH12.2H7	329918	BioLegend
ICOS	PE	ISA3	12-9948-42	e Bioscience
CD19	FITC	2185634	555412	BD Pharmingen
CD24	BV605	ML5	311124	BioLegend
CD27	BV510	L218	563090	BD Biosciences
CD27	APC	M-T271	356410	BioLegend
CD38	PerCP-Cy5.5	HB7	356613	BioLegend
CD69	FITC	FN50	310904	BioLegend
CD138	PeCy7	DL-101	352318	BioLegend
IgD	AF700	IA62	348229	BioLegend
IL-27Rα	PE	191106	FAB14791P-025	R&D Systems
BLIMP-1	APC	ACTU0116021	IC36081a	R&D systems
PD-1	BV510	EH12.2H7	329931	BioLegend
IL-4	PeCy7	MP4-25D2	500823	BioLegend
IL-17A	PE	BL168	512306	BioLegend
IL-21	AF647	3A3-N2	513005	BioLegend
IL-22	PeCy7	2G12A41	366707	BioLegend
IL-27p28	APC	307426	IC25261A	R&D Systems
IL-27EBI3	PE	B032F6	360904	BioLegend
IFN-γ	BV605	4S.B3	502536	BioLegend

### Multiplex Cytokine Bead Array Assay

Cytokines including IL-21, IL-27p28/EBI3, IFN-γ, IL-4, IL-17A, and IL-22 were detected in supernatant collected from sorted T_FH_ cells after 18 h of PMA/ionomycin stimulation. Levels of plasma cytokines were quantified by multiplex cytokine bead array assay as per the manufacturer’s instruction (Invitrogen). For data analysis, standard curve was derived using standards provided in the kit and conc. of each cytokine was calculated.

### Detection of Antibodies by ELISA

Antibodies including IgG (Cat no. BMS2091) IgM (Cat no. BMS2098) and IgA (Cat no. BMS2096) were analysed by using kits from Invitrogen. ELISAs were performed on both plasma as well as cell supernatant. Plasma samples were diluted as per the technical instructions; however, no dilutions were done for cell supernatant. Plasma and cell supernatant were loaded in duplicate wells and ELISA was performed as per the technical instructions provided in the kit. Absorbance was measured at 450 nm with reduction at 630 nm using an ELISA plate reader.

### Sorting of T_FH_, Naïve and Memory B Cells

For cell sorting, CHB and HC-vacc PBMCs were thawed, washed with pre-warmed complete RPMI1640 medium and rested overnight at 37° in CO2 incubator. The following day, cells were washed twice with 1× PBS and single suspensions were obtained. Cell viability was quantified by trypan blue exclusion assay and always showed >90% viability. PBMCs were stained with anti-human CD3 APC-Cy7, CD4 PerCP-Cy5.5 and CXCR5 BV421 to sort T_FH_ cells. For B cells, CD19 FITC, IgD AF700 and CD27 APC antibodies were used. Naive B cells were defined as CD19+IgD+CD27- cells. Memory B cells were sorted based on CD19+CD27+ cells. Cells were collected in 2 ml FBS. Purity of the sorted cells was measured and found ≥ 90%.

### Determination of Relative mRNA Expression by Quantitative Real-Time PCR (qRT-PCR)

Total RNA was isolated from sorted T_FH_ cells using RNeasy Plus mini kit (Qiagen, Cat no: 74134) as per the manufacturer’s instruction. RNA concentration was measured on a Nanodrop ND-1000 spectrophotometer (Thermo Fisher Scientific). cDNA was synthesized by reverse transcriptase polymerase chain reaction using random hexamer. SYBR Green qRT-PCR reaction was performed with 7500 Real Time PCR System (Applied Biosystems). The primers of selected gene were designed using Primer 3 software. Primers are as follows: 18s (Forward: AAGTACGCACGGCCGGTACA, Reverse: AGCGCCCGTCGGCATGTATT) IL-21 (Forward: TTCTGCCAGCTCCAGAAGAT, Reverse: TTGTGGAAGGTGGTTTCCTC) and IL-27 (Forward: CAGACGGCAGGCGACCTT, Reverse: GAGATGCAGGCTGACTGTGA). Gene expression level was normalized against 18S RNA (control gene). Subsequently, the relative gene expression values were determined using log of 2^−ΔΔCT^.

### Co-Culture of T_FH_ and B Cell Subsets

For T_FH_ and B cell co-culture experiment, T_FH_, naïve and memory B cells were sorted as per the above-mentioned protocol. Sorted T_FH_ cells were first primed with PepMix HBV surface peptides for 3 h (1 µg/ml) at 37^0^ in 5% CO2 incubator and subsequently washed with pre-warmed complete RPMI1640 medium to wash off HBsAg. Depending on the cell number achieved during cell sorting, 3-4 X 10^4^ T_FH_ cells were cultured with autologous memory and naïve B cells in 1:1 ratio for 5 days in the presence of anti-human IL-21 (1 µg/ml) (MT216G/21.3m Product code 3540-0N-500, MABTECH) and anti-human IL-27 (1 µg/ml) (Cat no. AF2526, R&D systems) neutralizing antibodies alone and in combination. At the end of co-culture, supernatant was harvested and stored at −80°C for IgG, IgM, and IgA detection. A PBS wash was given to the cells and then cells were stained with anti-human CD19, CD27, CD38, and CD138 monoclonal antibodies to observe the generation of plasmablasts and plasma cells. Intracellular staining was performed as per above mentioned protocol to investigate Blimp-1 expression. Prior studies indicate that plasmablasts and plasma cells lose the expression of CD19 (11, 19). Consistent with these previous data, our samples also showed extremely low percentage of CD19+ plasmablasts and plasma cells, therefore we considered CD27+CD38+ cells as plasmablasts and CD27+CD38+CD138+ population as plasma cells.

### B Cell Stimulation

T_FH_ cells produce several cytokines, therefore to confirm that T_FH_ mediated B cell help is through IL-27 but not any other cytokine, naïve and memory B cells were incubated with and without recombinant-human IL-27 (rIL-27) (Cat no. 589202, BioLegend) at a concentration of 100 ng/ml for 5 days and the generation of plasmablasts and plasma cells was analyzed.

### B Cell ELISpot Assay

B cell ELISpot assays were performed according to standard protocol established in our laboratory. In detail, after thawing, PBMCs were suspended in complete RPMI1640 medium and rested overnight in humidity at 37^°^C and 5% CO2 incubator. Next day, cells were washed twice, counted by trypan blue and subsequently cultured under following conditions (1) Control, where no stimuli was given (2) rIL-21 stimulation at a conc. of 100 ng/ml (Cat no. 571204, BioLegend) (3) rIL-27 stimulation (100 ng/ml) (4) R848+rIL-2 stimulation (1 µg and 10 ng, respectively, R848: Cat no. tlrl-r848, InvivoGen and IL-2: Cat no. 589102, BioLegend) (5) IL-21 stimulation in combination with R848+IL-2 (6) IL-27 stimulation in combination with R848+IL-2 (7) IL-21+IL-27+R848+IL-2 together for 5 days in CO_2_ incubator with 37°C humidity. A sterile 96-well multiscreen-IP filter ELISpot plate with a PVDF membrane (Cat no. MAIPSWU10, Mabtech) was activated with 70% ethanol and washed with ultrapure H_2_O. To measure HBV-specific response, wells were coated with recombinant HBsAg subtype adw (10 μg/mL, Cat no. 30R-AH016, Fitzgerald) diluted in 1× PBS (pH7.4). For total B cell antibody response, wells were coated with anti-human IgG, IgM and IgA (5 μg/mL, Cat no. 3860-4-250, 3850-3-250, 3880-3-250 respectively, Mabtech) for overnight at 4°C. The next day, the plate was washed with sterile PBS and blocked with 5% BSA/PBS for 1 h at 37°C incubator. After 5 days culture, PBMCs were washed twice with complete RPMI 1640 and cells were counted and 1 × 10^6^ cells were added into the top row of ELISpot for HBsAg-specific IgG, IgM and IgA, 25 × 10^3^ cells for total IgG, IgM and IgA and 1 × 10^5^ cells for controls, followed by 3-fold dilutions down to the bottom row. The plate was then incubated for 18 to 24 h at 37 °C and 5% CO_2_. Frequency of antibody-secreting cells was measured after washing with 1× PBS-Tween 20 followed by incubation with Biotin-SP-conjugated anti-human IgG, IgM and IgA (1:1000, Product code 3860-2HW-Plus Mabtech) (1:1000, Cat no. 709-066-149, 709-066-073, Jackson ImmunoResearch) for 1 h at room temperature, then AP-conjugated streptavidin (1:5000, Cat no. 710-04, Southern Biotech) for another 1 h. The spots were then developed for 3-5 minutes in dark using Vector blue-AP substrate kit (Cat no. SK-5300, Vector lab). The plate was air-dried overnight in the dark. An automated ImmunoSpot image analyzer (Cellular Technology Limited) was used to count the spots. Quality control was performed for the ultimate judgment on the quality of results and any non-specific spots were removed. Frequency of HBsAg-specific as well as total IgG, IgM and IgA antibody secreting B cells was calculated in each well. Data is represented as number of spots/10^6^ cells.

### Statistical Analysis

Statistical analyses were executed in GraphPad Prism 5. Data comparison was done using Mann-Whitney U test, paired or unpaired t-test as per the requirement. One-way analysis of variance (ANOVA) test and Kruskal-Wallis with Dunns multiple comparison test was performed for multiple comparisons. Correlation significance was calculated by Pearson equation. Data have been represented as median with range or mean with standard deviation. P value <0.05 was considered for significance.

## Results

Demographic and clinical profile of the patients and HC-vacc have been listed in **Table 2**.

**Table 2:** Demographic and clinical parameters of the study subjects.

Parameters	Hepatitis B vaccinated healthy controls (HC-vacc) (n = 26)	Chronic Hepatitis B (CHB) (n = 36)
Age (years)	42 (23–63)	49 (27–72)
Male: Female	1:1	1:1
ALT (U/L)	25 (23–32)	61 (22–193)
AST (U/L)	22 (17–30)	45 (17–146)
HBsAg positive (n)	NA	36
Anti-HBs positive (n)	26	None
HBeAg positive (n)	NA	9
HBeAb positive (n)	NA	22
Anti-HBc positive (n)	NA	36
HBV DNA (copies/ml)	NA	1.6 × 10^5^ (<20–1.7 × 10^8^)
Race (n)		
Asians	6	13
African Americans	14	19
White	6	4

Values are presented as median (range) and number.

ALT, Alanine aminotransferase; AST, Aspartate aminotransferase. NA, not applicable.

### Modulation of T_FH_ Cell Compartment in CHB Patients

**Figure 1:**
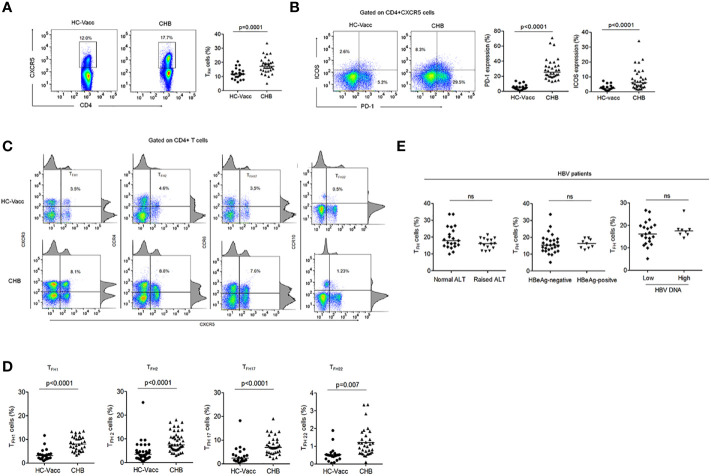
T_FH_ cell compartment was altered during chronic HBV infection. **(A, B)** Representative flow cytometry plot indicates the percent frequencies of circulating T_FH_ cells, PD-1 as well as ICOS expression in HC-vacc (n = 21) and CHB patients (n = 32) respectively. Throughout the flow cytometry plots, gates were placed based on single-color controls. **(C, D)** T_FH_ cell subtypes were analyzed on the basis of expression of different cell surface molecules. Values in the quadrant designate the percent frequency of different T_FH_ cell subsets. Cumulative data has been shown in scatter plot (HC-vacc: n = 21 and CHB: n = 32) and expressed as median. Each symbol on scatter plot represents an individual subject. Statistical analysis was performed using non-parametric, two-tailed Mann-Whitney *U* test. **(E)** Frequency of T_FH_ cells in HBV patients with different ALT and HBV DNA levels, HBeAg-negative and positive patients was analyzed. Levels of ALT were divided as normal (range, 22–40; n=21) vs. raised (range, 41–193; n=15)). HBV DNA levels were divided as <10^4^ (n = 22) and >10^4^ (n = 8). Unpaired t-test was used for statistical significance.

To determine whether a quantitative deficiency of circulating T_FH_ cells could contribute to lack of B cell help, we investigated the percentage of circulating T_FH_ cells in CHB patients and compared with HC-vacc. Gating strategy for T_FH_ cells has been shown in **Supplemental Figure 1A**. We found that the frequency of T_FH_ cells was increased in circulation of CHB patients than HC (**Figure 1A**), consistent with previous studies (20, 21). Because PD-1 is a third canonical marker of T_FH_ cells, we analyzed PD-1 expression and found it to be markedly elevated in CHB. Furthermore, ICOS expression, important for maintaining T_FH_ cell phenotype and function and further B cell antibody response, was significantly enhanced in CHB (**Figure 1B**). A study reported that blood CD4+CXCR5+ T_FH_ cells comprise different subset of T_FH_ cells that induce B cell antibody production (22); therefore, to analyze whether CHB infection make any changes in the frequencies and function of these subpopulations, we analyzed different subset of T_FH_ cells in CHB infected patients and compared with the HC-vacc. Different subset of T_FH_ cells including T_FH1_, T_FH2_, T_FH17_, and T_FH22_ were significantly increased in CHB (**Figures 1C, D**) which suggest that CHB infection alter the frequencies of these subsets. Additionally, varied CD4 T helper cell subsets comprising Th1, Th2, Th17 and Th22 were also higher in CHB patients and displayed greater PD-1 expression (**Supplemental Figures 1B, C**). These results demonstrate that altered T_FH_ cell function in CHB is not as a result of a decrease in T_FH_ cells.

Furthermore, to determine if T_FH_ cell frequency alters with change in clinical and viral measures, distribution of patients was done according to their ALT levels, HBeAg status and HBV DNA levels. The result showed similar frequency of T_FH_ cells in patients with normal and raised ALT, low and high HBV DNA levels and HBeAg-negative and positive patients (**Figure 1E**).

### In CHB, T_FH_ Cells Exhibited HBsAg-Specific IL-27 Expressing Cells, While Frequencies of IL-21 Expressing Cells Were Diminished

**Figure 2:**
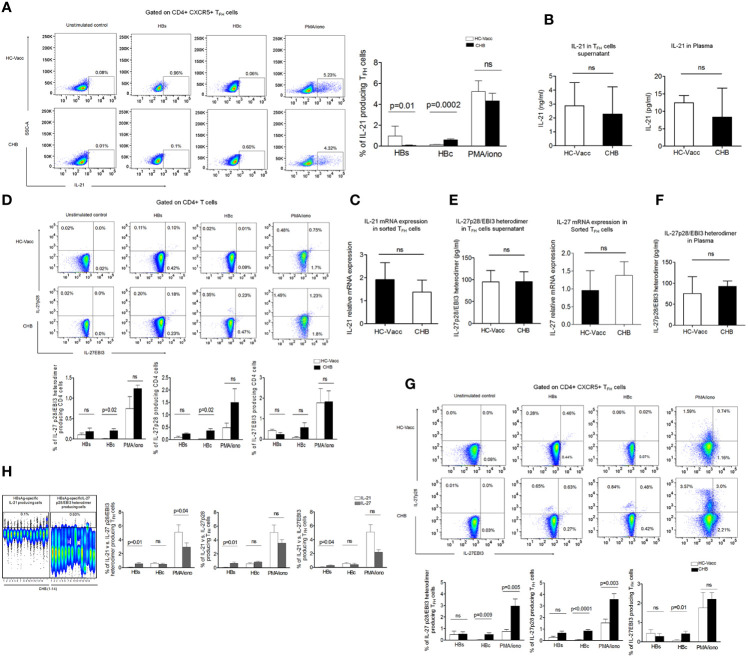
HBsAg dysregulate IL-21 secretion by T_FH_ cells but do not obstruct IL-27 production. **(A)** Representative flow cytometry plot and collective data in bar graphs illustrate HBV-specific IL-21 expressing T_FH_ cells after stimulation with PepMix HBV large envelop protein Ultra (HBs) which has a mix of 216 peptides (15mers with 11 aa overlap) and HBV capsid/core protein which has a pool of 44 peptides, at a concentration of 1 μg/ml for 5 days in the presence of CD49d and CD28 (2 μg/ml), re-stimulation with HBs and HBcAg was done on day 4. Global IL-21 expressing cells were measured after overnight PMA/ionomycin stimulation followed by intracellular cytokine staining and subsequent data analysis by flowJo. Cells without any stimulation were taken as controls. **(B)**. IL-21 was detected by multiplex cytokine bead array assay in cell supernatant collected after T_FH_ cell sorting and following stimulation with PMA/ionomycin as well as in plasma. **(C)** Analysis of IL-21 Relative mRNA expression in sorted T_FH_ cells **(D)** HBV-specific and global IL-27p28/EBI3, IL-27p28, and IL-27EBI3 producing cells were assessed by CD4 T cells after 5 days stimulation with HBs, HBc peptides and PMA/ionomycin as mentioned above. **(E)** IL-27p28/EBI3 heterodimer production was also evaluated specifically in sorted T_FH_ cells supernatant stimulated with PMA/ionomycin and relative mRNA expression was analyzed in sorted T_FH_ cells **(F)** IL-27p28/EBI3 heterodimer was also measured in the plasma **(G)** HBV-specific as well as global IL-27p28/EBI3, IL-27p28, and IL-27EBI3 producing T_FH_ cells directed against HBs and HBc peptides and PMA/ionomycin. **(H)** Comparison of IL-21 vs. IL-27p28/EBI3 heterodimer expressing T_FH_ cells in CHB patients; HBsAg-specific cytokine producing cells has been shown in representative concatenate flow cytometry plot and cumulative data has been demonstrated in bar graphs where IL-21 expressing T_FH_ cells were compared with IL-27p28/EBI3 heterodimer as well as IL-27p28 and IL-27EBI3. All the flow cytometry cytokine analysis was performed in fourteen CHB and HC-vacc in each group. To measure cytokine in sorted T_FH_ cells supernatant, four HC-vacc and three CHB patients were taken. Plasma cytokines were detected in nine CHB and HC-vacc in each group. Bars indicate median with range or mean with standard deviation. P values were determined either by non-parametric, 2-tailed Mann-Whitney *U* test or unpaired t test.

IL-21 is the signature cytokine of T_FH_ cells and known to be associated with antiviral response. Thus, we first analyzed the frequencies of HBV-specific as well as global IL-21 producing T_FH_ cells. For analyzing the frequency of cytokine expressing T_FH_ cells, gating strategy has been shown in **Supplementary Figure 2A**. Our data demonstrated that in CHB the frequencies of HBsAg-specific IL-21 expressing T_FH_ cells were lower in comparison to HC; while HBcAg-specific IL-21 producing T_FH_ cells were preserved. The frequency of global IL-21 producing T_FH_ cells remained comparable between CHB and HC-vacc (**Figure 2A**). Further to validate that only HBsAg-specific but not global IL-21 secretion is impaired, sorted T_FH_ cells were stimulated with PMA/ionomycin overnight, supernatant was collected, and IL-21 level was analyzed by multiplex cytokine bead array assay. No significant differences were seen in IL-21 levels between CHB and HC-vacc, Likewise, plasma IL-21 level remained comparable between CHB and HC-vacc (**Figure 2B**). Moreover, no significant change in IL-21 relative mRNA expression was seen between CHB and HC (**Figure 2C**), suggesting that global IL-21 secretion is intact, whereas HBsAg-specific IL-21 production is impaired in CHB.

Previous study suggests that IL-27 act on CD4+T cells and facilitate T_FH_ differentiation (18)., this prompted us to evaluate whether CD4+ T cells itself secrete IL-27 or not. As IL-27 consists of two different subunits p28 and EBI3, we checked both subunits by flow cytometry. Surprisingly, CD4+T cells showed IL-27p28 and IL-27EBI3 producing cells after HBs, HBc and PMA/ionomycin stimulation (**Figure 2D**). Further, to specifically examine whether T_FH_ cells produce IL-27, supernatant from sorted T_FH_ cells was collected after overnight PMA/ionomycin stimulation and IL-27p28/EBI3 heterodimer was detected. Interestingly, our data revealed that T_FH_ cells efficiently secrete IL-27, both in CHB and HC-vacc. Moreover, to validate, we also analyzed the relative mRNA expression of IL-27 in sorted T_FH_ cells and found that T_FH_ cells expressed IL-27 mRNA in CHB as well as HC (**Figure 2E**). In addition, plasma IL-27 level was similar between CHB and HC-vacc. (**Figure 2F**). Moreover, to confirm whether T_FH_ cell population contain HBV-specific IL-27 producing cells, HBs and HBcAg-specific stimulations were performed. We found that T_FH_ cells contain HBV-specific IL-27 producing T_FH_ cells. HBsAg-specific IL-27p28 and IL-27EBI3 producing T_FH_ cells were comparable between CHB and HC-vacc individuals, whereas HBcAg or PMA/ionomycin induced IL-27 producing T_FH_ cells were more in CHB than HC-vacc (**Figure 2G**). Moreover, we compared IL-21 and IL-27 producing T_FH_ cells in CHB and observed that CHB patients have more HBsAg-specific IL-27 producing T_FH_ cells in comparison to IL-21 producing cells. Interestingly, HBcAg-specific IL-21 and IL-27 producing T_FH_ cells were comparable; while global IL-21 producing T_FH_ cells were higher in CHB (**Figure 2H**). Collectively, our data suggest that only HBsAg-specific IL-21 production is impaired in CHB.

As our data demonstrated a noticeable increase in different subsets of T_FH_ cells including T_FH1_, T_FH2_, T_FH17_ and T_FH22_, we evaluated related cytokines IFN-γ, IL-4, IL-17A, and IL-22 producing cells. HBsAg-specific IFN-γ and IL-22 producing cells were lower, while IL-17A producing cells were higher in CHB than HC-vacc. IL-4 producing cells were comparable between CHB and HC-vacc. HBcAg-specific and global IFN-γ, IL-4, IL-17A and IL-22 producing cells were elevated in CHB than HC-vacc (**Supplemental Figures 2B, C**). Additionally, secretion of global IFN-γ, IL-4, IL-17A, and IL-22 was measured in sorted T_FH_ cells supernatant as well as plasma and appeared slightly higher in CHB compared to HC-vacc, (**Supplemental Figures 2D, E**), except for IL-22 which could not be detected in the plasma of CHB patients.

### PD-1^+^ T_FH_ Cell Population Majorly Contribute to IL-27 Secretion

**Figure 3:**
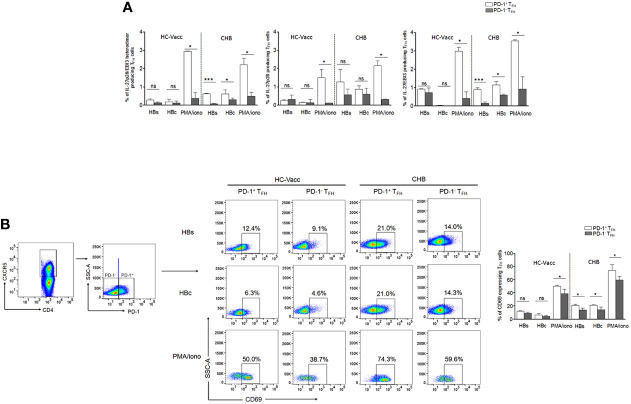
HBV-specific as well as global IL-27 expressing T_FH_ cells were augmented in PD-1^+^ fraction of cells rather than PD-1^-^. **(A)** HBV-specific as well as global cytokine producing T_FH_ cells were assessed in PD-1^+^ and PD-1^-^ T_FH_ cell population in CHB and HC-vacc (n=14 subjects in each group). **(B)** To identify the functional status of PD-1^+^ T_FH_ cells, expression of CD69, an activation marker, was analyzed on both PD-1^+^ and PD-1^-^ cells after HBs, HBc and PMA/ionomycin stimulation. Statistics were calculated using 2-tailed non-parametric, Mann-Whitney *U* test. Bars graphs indicate median with range. * indicates p < 0.05 and ***p < 0.001.

Our data revealed that T_FH_ cells produce IL-27, therefore, to find out which T_FH_ cell population contribute to high IL-27, we determined IL-27 producing cells in PD-1^+^ and PD-1^-^ T_FH_ cell population. We found that particularly in CHB, PD-1^+^ T_FH_ cells majorly contain HBV-specific and global IL-27 producing cells rather than PD-1^-^ population (**Figure 3A**). In addition, we examined other T_FH_ cell cytokine producing cells in PD-1^+^ and PD-1^-^ T_FH_ population. We observed that HBV-specific and global IL-21, IFN-γ, IL-4 and IL-22 producing cells were evidently higher in PD-1^+^ population rather than PD-1^-^ cells in CHB, except for IL-17A producing cells which were comparable between both populations. In case of HC-vacc, PD-1^+^ fraction of T_FH_ cells have higher HBsAg-specific IL-21– and IL-22–producing cells. Global IL-21, IFN-γ, IL-4 and IL-22 producing cells were also higher in PD-1^+^ fraction of cells (**Supplemental Figure 3**). This clearly demonstrates that particularly in CHB, HBV-specific PD-1^+^T_FH_ cells are functionally activated. Moreover, we verified activation status of PD-1^+^ T_FH_ by analyzing CD69 (activation marker) expression after HBs, HBc and PMA/ionomycin stimulation (**Figure 3B**). CHB patients showed higher CD69 expression on PD-1^+^T_FH_ cells than PD-1^-^ population that reiterates our assumption that PD-1^+^ T_FH_ cells are activated but not exhausted. Our data is also supported by previous studies, which describes PD-1 as an activation marker for T_FH_ (23, 24).

### B Cell Phenotype Was Altered in CHB Patients With Increased Blimp-1 and IL-27R Expression

**Figure 4:**
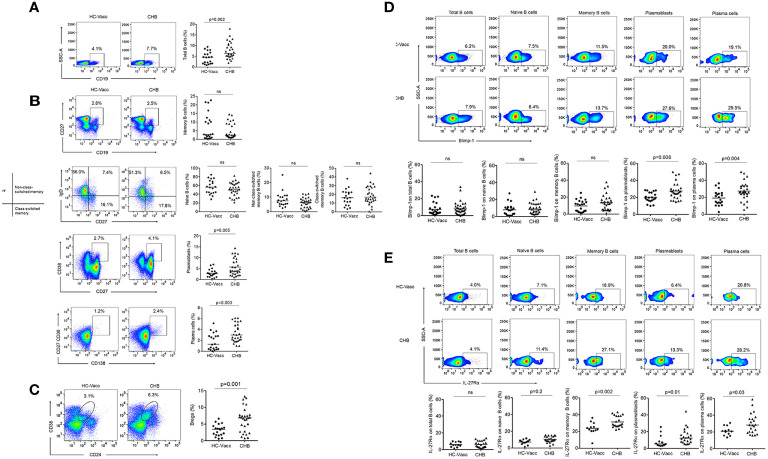
Modification in B cell during chronic HBV infection**. (A–C)** Percent frequencies of CD19+ B cells, memory, non-class-switched, class-switched, naïve, plasmablasts, plasma cells and regulatory B cells were analyzed by flow cytometry, represented in flow cytometry plot and collective data in scatter plot (CHB: n=29, HC-vacc: n=20) **(D)** Expression of Blimp-1 (CHB: n=29, HC-vacc: n=20) **(E)** IL-27Rα was analyzed on different subset of B cells (CHB: n=22, HC-vacc: n=12). Data represented as median. Significance was calculated by non-parametric, 2-tailed Mann-Whitney *U* test.

To test whether frequencies of total as well as different subset of B cells are abnormal during CHB, we comprehensively evaluated B cells. Percentage of CD19+ B cells was significantly increased in CHB than HC-vacc while memory (CD19+CD27+), non-class-switched (CD19+CD27+IgD+), class-switched (CD19+CD27+IgD-) and naive (CD27-IgD+) B cells were comparable. Plasmablasts (CD27+CD38+), plasma cells (CD27+CD38+CD138+) and immune-suppressor B cells, termed as regulatory B cells (Bregs) (CD19+CD24+CD38+) that modulate immune response by suppressing the proliferation and cytokine production of effector T cells (25), were significantly increased in CHB (**Figures 4A–C**).

In B cell lineage, Blimp-1 is critical for the development of immunoglobulin secreting cells and maintenance of long-lived plasma cells; therefore, we examined Blimp-1 expression. Total, naïve and memory B cells did not show any alteration in Blimp-1 expression among CHB patients and HC-vacc, but plasmablasts and plasma cells showed higher Blimp-1 expression in CHB (**Figure 4D**).

IL-27 mediated B cell signaling require its binding to a heterodimeric receptor comprising of ligand-specific IL-27Rα chain and gp130, shared with many other cytokines including IL-6. Despite, shared use of the gp130 chain, the influence of IL-27Rα subunit makes IL-27 functionally distinct from IL-6. Therefore, we specifically determined the expression of IL-27Rα on B cells. Our findings indicate that B cell subsets express IL-27Rα that was significantly higher in CHB (**Figure 4E**). When different B cells subsets were compared with respect to IL-27R expression, memory B and plasma cells expressed highest IL-27R; while total B, naïve B and plasmablasts showed lower IL-27R expression.

### Correlation Between IL-27p28/EBI3+ T_FH_ Cells With B Cells, Plasma Antibodies, Level of HBV DNA, and Markers of Liver Inflammation

**Figure 5:**
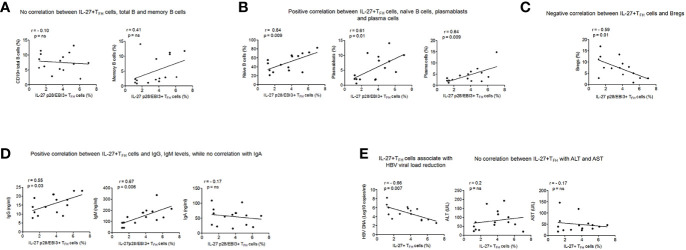
Association of IL-27 p28/EBI3+ T_FH_ cells with B cell subsets, plasma antibodies, levels of HBV DNA and marker of liver inflammation **(A)** Correlation between IL-27 p28/EBI3+ T_FH_ cells, total and memory B cells **(B)** between IL-27 p28/EBI3+ T_FH_ cells, naïve B cells, plasmablasts and plasma cells **(C)** between IL-27 p28/EBI3+ T_FH_ cells and Bregs **(D)** levels of plasma antibodies **(E)** IL-27 p28/EBI3+ T_FH_ cells had significant association with decreased HBV viral load, while no correlation was seen with ALT and AST levels.

We next asked whether IL-27p28/EBI3+ T_FH_ cells had any association with B cells, levels of plasma antibodies, HBV DNA and markers of liver inflammation in CHB. Our results indicated no significant association between IL-27p28/EBI3+ T_FH_ cells with total (r = 0.10) and memory B cells (r = 0.41) (**Figure 5A**), however a positive correlation was seen with naïve B cells (r = 0.64, p = 0.009), plasmablasts (r = 0.61, p = 0.01) and plasma cells (r = 0.64, p = 0.009) (**Figure 5B**). A negative correlation was seen between IL-27p28/EBI3+ T_FH_ cells and Bregs (r = −0.59, p = 0.01) (**Figure 5C**). Importantly, IL-27p28/EBI3+ T_FH_ cells showed significant positive association with the levels of plasma IgG (r=0.55, p=0.03) and IgM (r=0.67, p=0.006), while no correlation was seen with IgA (r = −0.17) (**Figure 5D**).

High HBV DNA is considered major contributing factor in the development of cirrhosis, and further HCC; therefore, we analyzed the association between IL-27p28/EBI3+ T_FH_ with HBV DNA. We observed a significant negative correlation between IL-27p28/EBI3+ T_FH_ and HBV DNA (r= -0.66, p=0.007). In clinical practice, ALT and AST are the common index to reflect the liver damage. Hence, we studied the association between IL-27p28/EBI3+ T_FH_ cells and markers of liver injuries and we did not find any correlation (ALT: r=0.2, AST: r=-0.17) (**Figure 5E**). These results demonstrate that IL-27p28/EBI3+ T_FH_ cells may constrain HBV DNA level and are not involved in the pathological process of HBV infection and related liver injury.

### T_FH_ Cells Support Plasmablasts and Plasma Cell Formation Through IL-27

Several studies demonstrate that T_FH_ cell facilitates B cell help through IL-21, and reduction in IL-21 could lead to defective B cell response. However, T_FH_ mediated B cell support has been shown to still occur despite low levels of IL-21 (11). It is most likely that some anonymous molecule is contributing in accomplishing dynamic B cell functions. We hypothesize that T_FH_ cells produce IL-27, which compensate the function of IL-21 and support B cell functions. To prove, we performed T_FH_ and B cell co-culture. To mimic the antigen-specific cytokine mediated interaction between T_FH_ and B cells, sorted T_FH_ cells were first primed with HBsAg for 3 h, washed and then incubated with autologous memory and naïve B cells from CHB patients and HC-vacc for 5 days in the presence and absence of IL-21 and IL-27 neutralizing antibodies. The antibody secreting cell compartment consists of short-lived proliferating plasmablasts and long-lived plasma cells. Thus, we investigated the generation of both plasmablasts and plasma cells. T_FH_ cells induced memory and naïve B cells to become plasmablasts and plasma cells. Memory B cells were more differentiated into plasmablasts and plasma cells than naive B cells. Notably, neutralization of IL-27 significantly restricted plasmablasts and plasma cell formation from memory and naïve B cells compared to the control where no blockade was done. These results signify the potential contribution of IL-27 in plasmablasts and plasma cell differentiation. Of note, neutralization of IL-21 did not considerably inhibit the generation of plasmablasts and plasma cells in CHB. This could be because CHB patients already had minimal HBsAg-specific IL-21 secretion, thus neutralization would not affect IL-21 mediated B cell response substantially.

**Figure 6:**
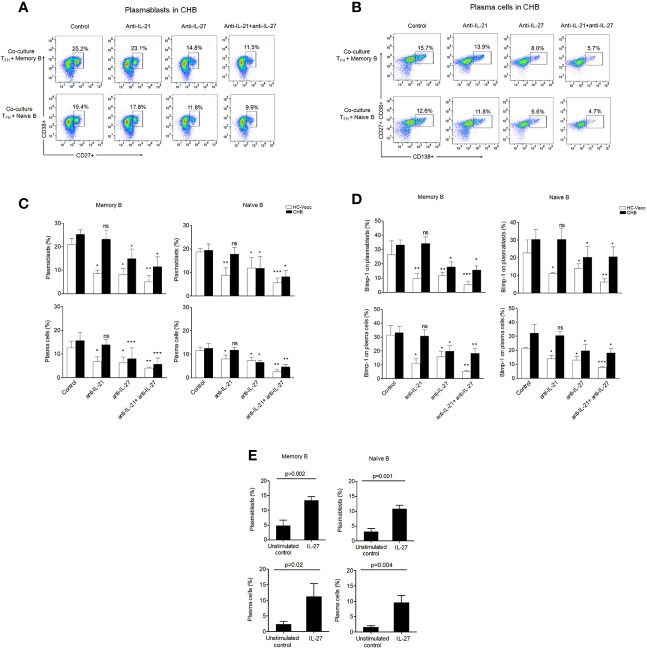
IL-27 secreted by T_FH_ cells support plasmablasts and plasma cell formation. **(A–C)** Mechanism of T_FH_ mediated B cell response was determined by autologous T_FH_-B cell co-culture (CHB: n=5, HC-vacc: n=5). To imitate HBV-specific interactions between T_FH_ and B cells, FACS-sorted T_FH_ cells were first primed with HBsAg for 3 h, washed and then incubated with autologous CD19+CD27+ memory and CD19+CD27-IgD+ naïve B cells with and without of IL-21 and IL-27 neutralizing antibodies for 5 days. Generation of plasmablasts and plasma cells was analyzed by flow cytometry **(D)** expression of Blimp-1 (CHB: n=5, HC-vacc: n=5). Plasmablasts were gated as CD27+CD38+ cells and plasma cells were defined based on CD27+CD38+CD138. **(E)** Incubation of memory B and naïve B cells with rIL-27 for 5 days showed increased plasmablasts and plasma cell formation (CHB: n=5). Statistical analysis was performed using either Kruskal-Wallis test (ANOVA) with Dunn’s *post hoc* test for multiple comparisons or paired t test. Bars indicates mean and error bars designate standard deviation. * indicates p < 0.05, **p < 0.01 and ***p < 0.001.

In HC-vacc, similar data was obtained with regard to IL-27; however, neutralization of IL-21 markedly inhibited plasmablasts and plasma cell formation (**Figures 6A–C**). These data collectively demonstrate that in the absence of IL-21 secretion in CHB, T_FH_ cells help B cell through IL-27.

Since, Blimp-1 is critical for Immunoglobulin secretion (26); hence, the effect of IL-27 neutralization on Blimp-1 was analyzed. Our results showed significant reduction in Blimp-1 expression after IL-27 nullification, suggesting IL-27 as a potential inducer for Blimp-1. Moreover, in CHB, neutralization of IL-21 did not decrease Blimp-1 expression; however, HC-vacc showed a significant decline in Blimp-1 after IL-21 neutralization (**Figure 6D**).

To validate that T_FH_ derived B cell help is through IL-27, we performed another experiment where naïve and memory B cells were incubated in the presence and absence of rIL-27 for 5 days and plasmablasts and plasma cell formation was assessed. Stimulation of memory and naive B cells with rIL-27 immensely supported differentiation into plasmablasts and plasma cells as compared to the control (**Figure 6E**) which further supports the importance of IL-27 in B cell differentiation. Taken together, these data indicate that IL-27 not only supports plasmablasts and plasma cell formation but also induces Blimp-1 expression.

### IL-27 Induces HBsAg-Specific and Total Antibody Secretion by B Cells

**Figure 7:**
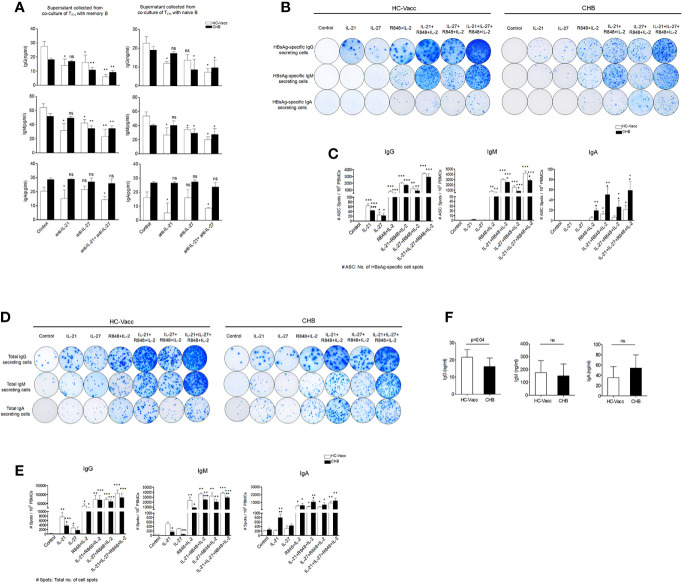
IL-27 help B cells for HBsAg-specific and total antibody secretion. **(A)** FACS-purified T_FH_ cells were first primed with HBsAg for 3 h, washed and then cultured with autologous memory and naïve B cells in the presence and absence IL-21 and IL-27 neutralizing antibody for 5 days. Supernatant was harvested and IgG, IgM and IgA were quantified by ELISA (CHB: n=5, HC-vacc: n=5). **(B-E)** Representative ELISpot image indicate HBsAg-specific as well as total IgG, IgM and IgA secreting cells in HC-vacc and CHB patients after 5 days culture in the presence of rIL-21, rIL-27, R848+IL-2 alone and in different combinations. No stimulation was given in controls. Bar graphs shows cumulative data of six subjects in each group and indicate mean of the no. of antigen specific as well as total antibody producing cell spot. P values were determined by Kruskal-Wallis test (ANOVA) with Dunn’s *post hoc* test for multiple comparisons or paired t test. Bars represent mean with standard deviation. Data comparisons were made between controls (without any stimulation) of HC-vacc and CHB with different stimulations and p values are flagged with *. Comparisons between HC-vacc and CHB were also analyzed and p values are flagged with ^♦^. *^,♦^ indicates p<0.05, **^,♦♦^ p<0.01 and ***^,♦♦♦^ p<0.001. **(F)** Levels of plasma antibodies were determined by ELISA and significance was calculated by non-parametric, 2-tailed Mann-Whitney *U* test.

Antibody production is an essential part of the B cell mediated response, providing both instant protection against an ongoing infection and long-term immunity. Consequently, we examined the role of IL-27 in IgG, IgM and IgA antibody production. To prove, T_FH_ cells were co-cultured with memory and naïve B cells in the presence and absence of IL-27 neutralizing antibodies for 5 days and then supernatant was collected for ELISA. Results showed a marked reduction in IgG and IgM production after IL-27 neutralization in CHB and HC-vacc; however, IgA did not change. Nullification of IL-21 significantly abolished IgG, IgM and IgA secretion in HC-vacc, but not in CHB (**Figure 7A**).

**Figure 8:**
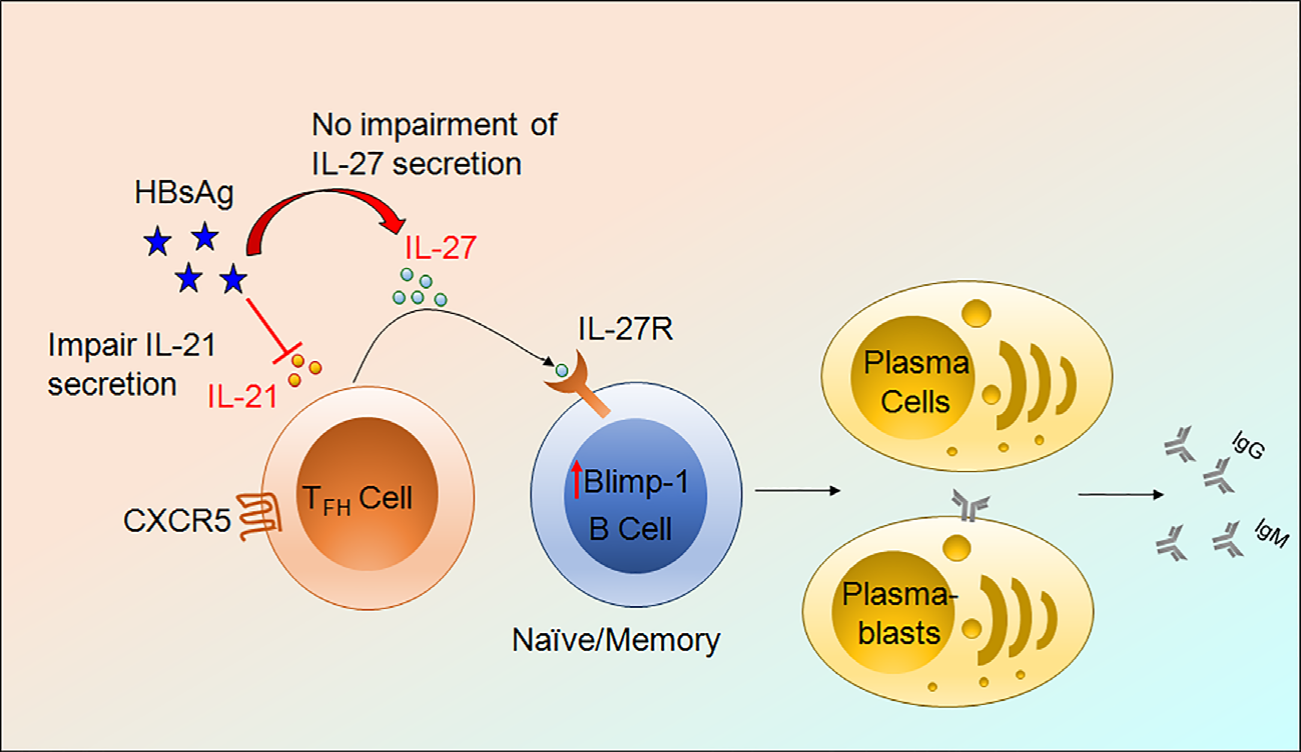
Mechanism of T_FH_ mediated B cells help. HBV infection impair IL-21 production by T_FH_ cells without obstructing IL-27 secretion. IL-27 binds to its cognate receptor present on the surface of B cells, activate downstream signaling, and further induces Blimp-1 expression and lead to the formation of plasmablasts and plasma cells resulting in HBsAg-specific and total antibody secretion.

Finally, to find out the effect of IL-27 on B cell antibody secretion, we performed HBsAg-specific and total ELISpot assay. Surprisingly, IL-27 enhanced HBsAg-specific and total IgG and IgM secretion in CHB and HC-vacc when used in combination with R848+IL-2; however, IgA secretion did not change. Stimulation with IL-27 alone improved HBsAg-specific and total IgG and IgM secretion in CHB individuals. In HC-vacc, stimulation with IL-27 alone induced only HBsAg-specific IgG secretion but not IgM and IgA; however, total antibody production was enhanced. HBsAg-specific and total antibody production was highest in cells stimulated with IL-21+IL-27+R848+IL-2 together. Moreover, in comparison to HC-vacc, CHB patients showed lower IgG and IgM, whereas IgA secretion was higher (**Figures 7B–E**). In addition, plasma IgG level was significantly lower in CHB patients than HC-vacc (**Figure 7F**). Together, our data showed that IL-27 production by T_FH_ cells lead to HBsAg-specific and total B cell response in CHB patients, which could help in viral clearance. Overall mechanism of T_FH_ mediated B cell help is described in **Figure 8**.

## Discussion

We report a novel mechanism of T_FH_ mediated B cell help through IL-27. Existing literature demonstrate that T_FH_ cells perform their functions *via* IL-21 which play an essential role in B cell activation, expansion, plasma cell generation and antibody production (10, 11). However, here we establish that, in CHB, regardless of severely impaired HBsAg-specific IL-21 production by T_FH_ cells, these cells are competent in supporting B cell functions by secreting IL-27 that support B cell response in terms of plasmablasts and plasma cell formation as well as protective antibody secretion. IL-27 not only improved total IgG and IgM production but also augmented HBsAg-specific antibody secretion by enhancing Blimp-1 expression. In addition, we observed a significant positive association between IL-27p28/EBI3+ T_FH_ cells with the expansion of naïve B cells, plasmablasts, plasma cells and related antibodies IgG and IgM. Moreover, we find that IL-27p28/EBI3+ T_FH_ cells correlate with reduction in HBV DNA, but not with markers of liver inflammation. During chronic HBV infection, live injury considered to be immune mediated, therefore the finding that the frequencies of IL-27p28/EBI3+ T_FH_ cells associate with lower HBV DNA level but not markers of liver inflammation suggest that IL-27p28/EBI3+ T_FH_ cells may help in HBV viral load reduction without contributing in the pathological process of HBV infection and related liver injury.

HBV-specific T cell response is associated with viral clearance; however, emerging evidence support a vital role of B cells in the immune control of HBV (27). Humoral antibodies against HBs and HBeAg are crucial for viral clearance and provides critical target for designing new immunotherapies and B cells are regarded as the source of protective antibodies. CHB patients do have HBsAg-specific B cells but with defective anti-HBs antibody production. Both HBsAg-specific and global B cell compartment have accumulation of atypical memory B cells with high expression of FcRL5 and PD-1 and these cells have altered signaling, homing, differentiation into antibody producing cells, resulting in impaired B cell immunity (28). In fact, our group reported the abnormal expansion of atypical memory B cells in CHB patients (29). However, the status of other B cell subset during CHB infection have not been extensively studied. Here, we *ex vivo* evaluated different subset of B cells and revealed that plasmablasts and plasma cells were increased and expressed higher Blimp-1 in CHB. Furthermore, in line with our *ex vivo* B cell analysis data, T_FH_-B co-culture data also confirmed the generation of more plasmablasts and plasma cells from memory and naïve B cells in CHB patients.

Until now, primarily macrophages and dendritic cells (30–34) have been reported as the main source of IL-27. A recent study demonstrated that malaria-specific CD4+ T cells produce IL-27 and regulate protective immunity during malaria parasite infection (35). Our study revealed that T_FH_ cells produce IL-27 during CHB infection. IL-27 is a heterodimeric cytokine which belongs to IL-12 cytokine family and consists of two protein subunit p28 (α chain) and Epstein-Barr virus-induced gene 3 (EBI3) (β chain) and signals through IL-27 receptor, formed by the association of IL-27Rα(also designated WSX-1 or TCCR) and gp130. IL-27 possesses antiviral functions against HBV (21),HCV (36) and HIV infection (37–39). The antiviral activity encouraged by the induction of signal transducer and activator of transcription (STAT)1 and STAT3 leading to the stimulation of IFN regulated proteins (21). Increased IL-27 level in CHB (40, 41) modulate immune response, prevent from hepatic injury (42) and critically involved in Th17 cell commitment exerting pro-inflammatory response (43, 44). IL-27 has also been demonstrated as a predictor of spontaneous HBeAg seroconversion (45).

Further, we focused on the potential role of IL-27 in B cell antibody production. We reported critical role of IL-27 not only in total IgG and IgM secretion but also in HBsAg-specific antibody production when used in combination with standard B cell stimuli R848+IL-2. Results of our study also supported by previous data showing the production of IgG1 antibody from human B cells in response to IL-27 (46). Similarly, exogenous supply of IL-21 also enhanced both HBsAg-specific and total IgG and IgM secretion both in CHB and HC-vacc when used in combination with R848+IL-2. Combination of IL-21 with IL-27 further boosted antibody secretion by B cells. Our data showed that B cells of CHB patients are responsive towards exogenous IL-21 and produce HBV-specific antibodies in response to IL-21, suggesting that low HBsAg-specific IL-21 level in CHB is partly accountable for reduced B cell antibody production. Although, our both *ex vivo* and *in vitro* data showed higher frequency of plasmablasts as well as plasma cells in CHB, yet HBsAg-specific and total antibody secretion was lower in comparison to HC-vacc, suggestive of partial functional impairment of B cells in these patients. It is important here to note that stimulation of B cells with IL-27 did not induce IgA production, this finding has significance. Constrained IL-27 mediated IgA production could be favorable for CHB patients as recent study reported the association of high serum IgA level with cirrhosis (47) and served IgA as an independent and potential biomarker for cirrhosis (48). Hence, IL-27 is likely to mediate a physiological response by skewing antibody secretion to a favorable IgG and IgM rather than IgA response.

Recently, it has been demonstrated that dysregulated T_FH_ cell response to HBsAg is due to the presence of high Tregs which inhibit HBV-induced T_FH_ and germinal center responses and promote HBV persistent by delaying HBsAg seroconversion (49). Here, we propose that continuous HBsAg exposure resulted in dysregulated T_FH_ response by selectively constraining IL-21 secretion but not IL-27 and suggests partial but not complete T_FH_ cell dysfunction in CHB.

Collectively, our data provide important insights into the novel mechanism of T_FH_ mediated B cell help. We established that IL-21 deficient T_FH_ cell help B cell through IL-27 and suggest that IL-27 could potentially compensate for most of the function of IL-21 which is evidenced with the fact that in spite of reduced HBsAg-specific IL-21 production in CHB, T_FH_ cells efficiently supported B cell functions. CHB patients still have defective humoral response and unlikely to achieve resolution. Hence, factors other than T_FH_ help are likely involved in complete reconstitution of effective B cell immune function in CHB patients. Future research is focused on unravelling additional intrinsic defects in development of effective and protective HBV specific humoral immunity in CHB patients.

## Data Availability Statement

The original contributions presented in the study are included in the article/**Supplementary Material**. Further inquiries can be directed to the corresponding author.

## Ethics Statement

The studies involving human participants were reviewed and approved by Institutional review board of the University of Maryland. The patients/participants provided their written informed consent to participate in this study.

## Author Contributions

AK conceived and designed the study. SK helped in study design and supervised the study. AK performed the experiments, generated, analyzed, and interpreted data, performed statistical analysis, and drafted manuscript. LT provided the patient samples and edited the manuscript. NA helped in generating ELISPOT data. BP commented on the manuscript. SK critically reviewed the manuscript. All authors contributed to the article and approved the submitted version.

## Funding

This study was conducted by department funds of the institute.

## Conflict of Interest

SK has received research grants paid to the university from Gilead Sciences, Merck and Arbutus pharmaceuticals.

The remaining authors declare that the research was conducted in the absence of any commercial or financial relationships that could be construed as a potential conflict of interest.

## Abbreviations

CHB, Chronic hepatitis B; CXCR, Chemokine receptor; HBV, Hepatitis B virus; HBsAg, Hepatitis B surface antigen; HBcAg, Hepatitis B core antigen; IL, Interleukin; Ig, Immunoglobulin; Blimp-1, B lymphocyte-induced maturation protein-1; T_FH_, T follicular helper; PMA, Phorbol 12-myristate 13-acetate; PD-1, Programmed death-1.
