# Supplementary material for: IL-21–Deficient T Follicular Helper Cells Support B Cell Responses Through IL-27 in Patients With Chronic Hepatitis B
Source: Front Immunol. 2021 Jan 28;11:599648. doi: 10.3389/fimmu.2020.599648 (PMC7876309; doi:10.3389/fimmu.2020.599648)
Supplement: Supplementary file 1 [file DataSheet_1.docx]

**Supplementary** **Information**

**Supplemental Figure 1A**


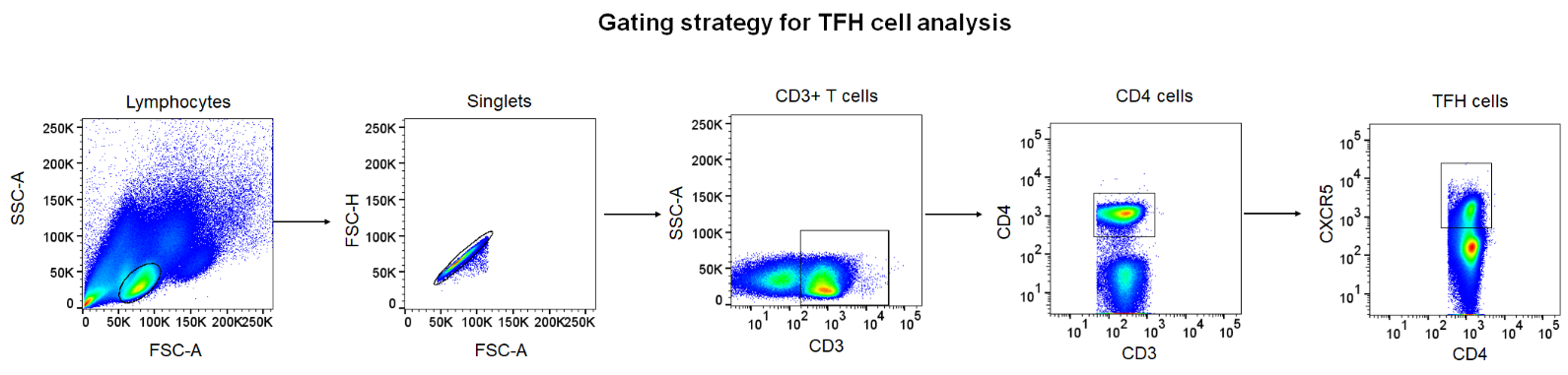


**Supplemental Figure 1B**


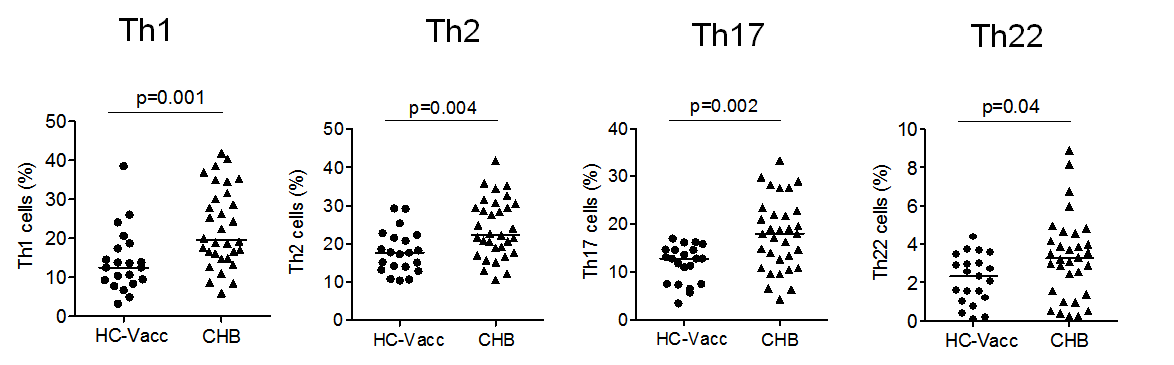


**Supplemental Figure 1C**


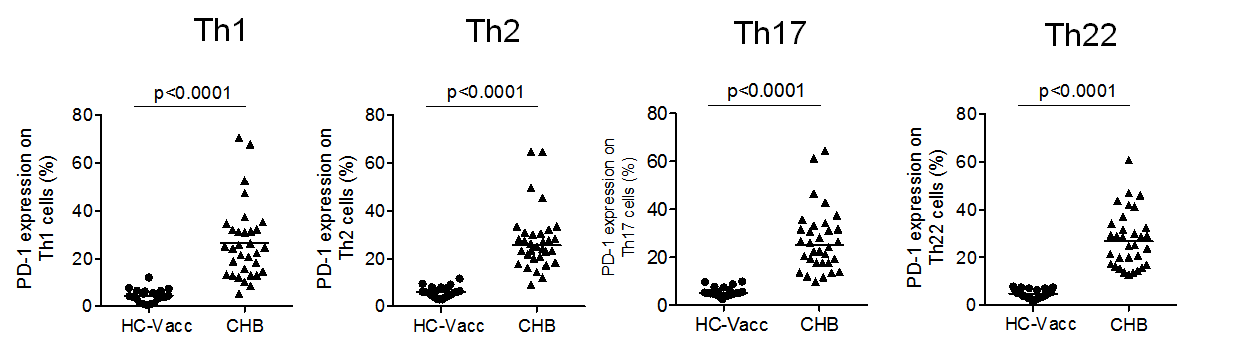


**Supplemental Figure 1**. **Different T helper cell subsets were increased in CHB patients and expressed high PD-1.** (A) Gating strategy for T_FH_ cell analysis (B, C) Frequencies of T helper cell subsets and expression of PD-1 was determined by flow cytometry. Significance was calculated by non-parametric, 2-tailed Mann-Whitney *U* test and scatter plots indicate median with range.

**Supplementary Figure 2A**


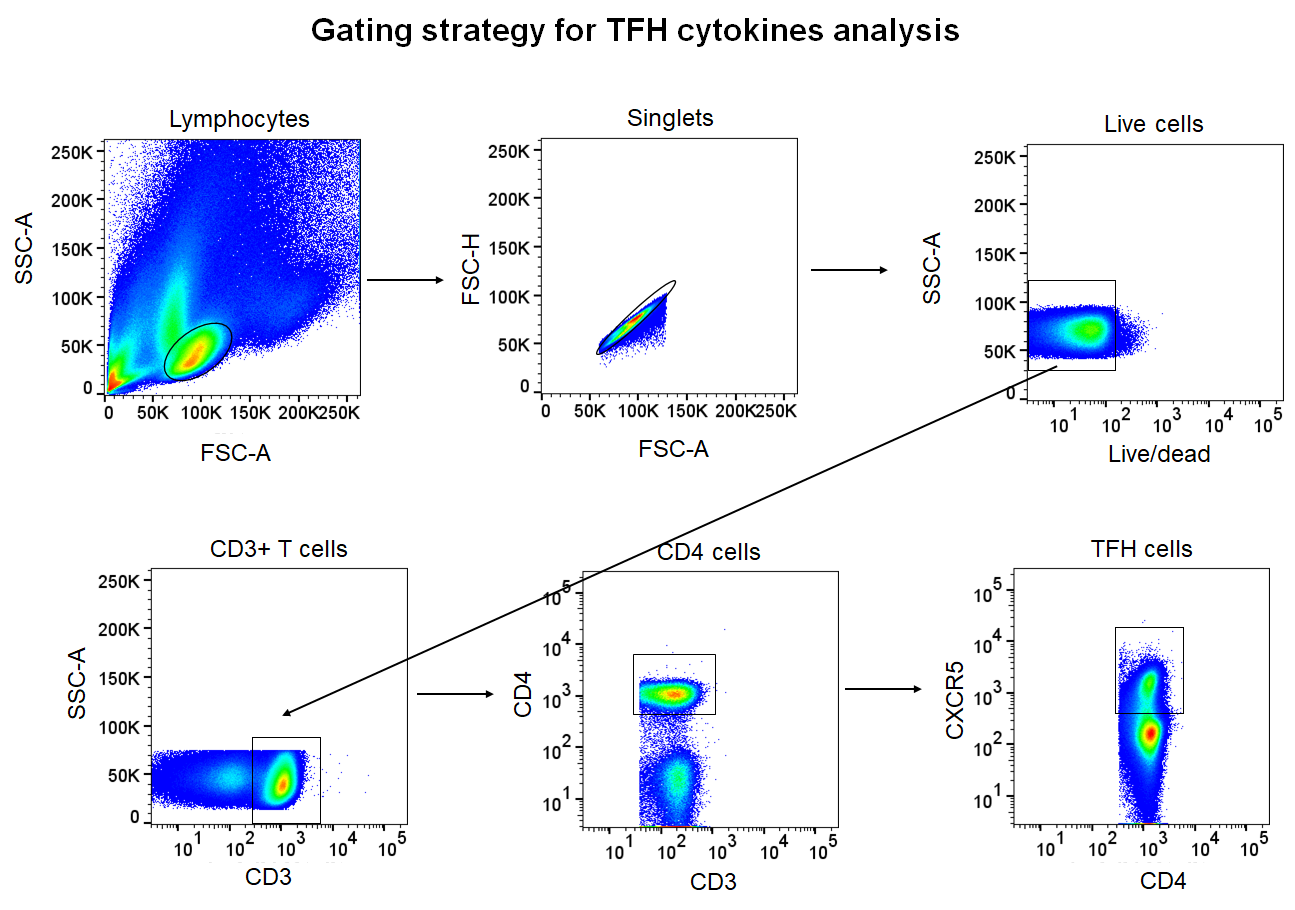


**Supplemental Figure 2C**

**Supplemental Figure 2B**


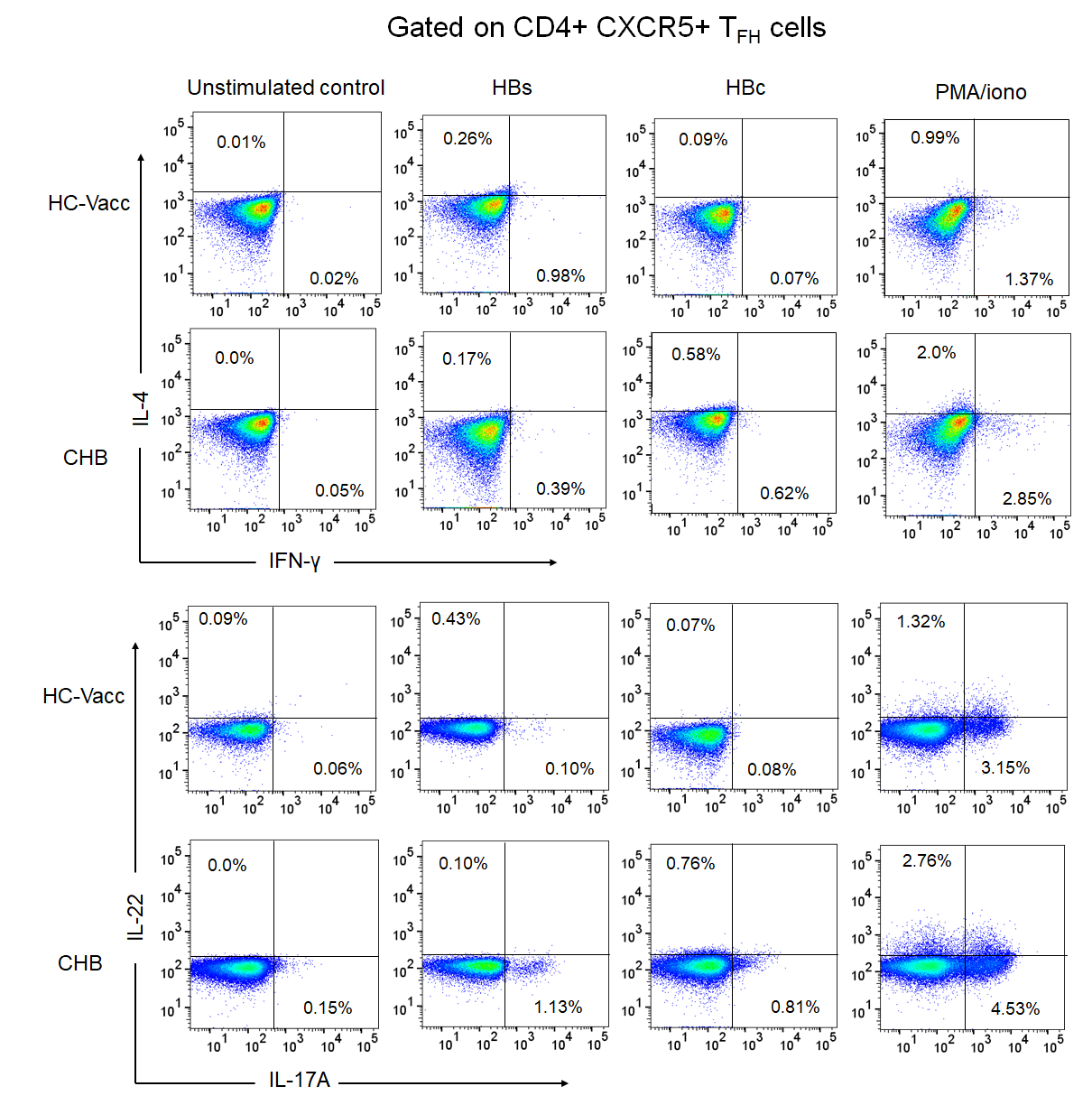


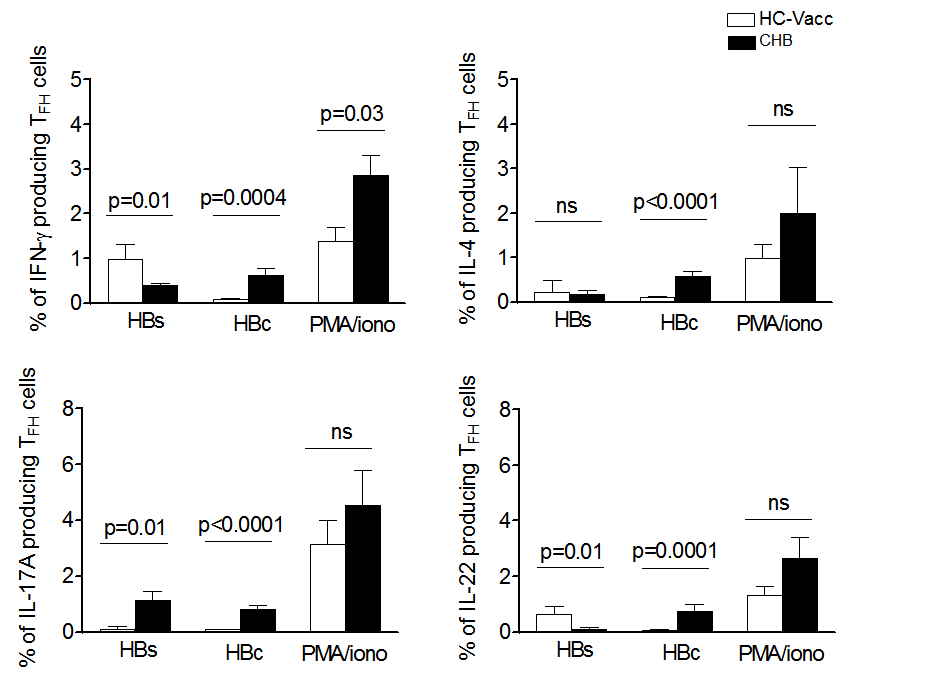


**Supplemental Figure 2E**

**Supplemental Figure 2D**


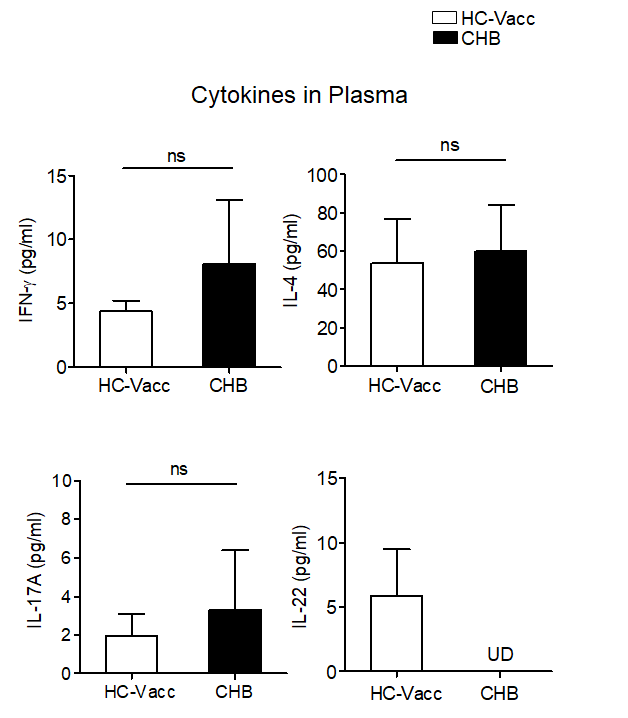


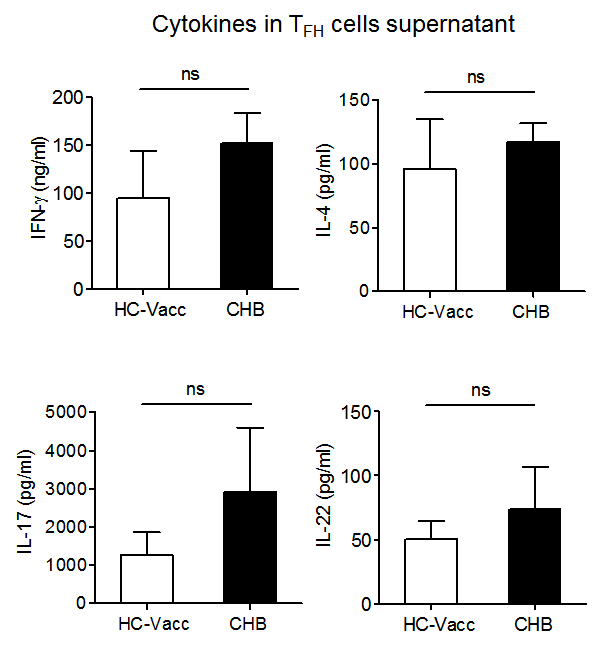


**Supplemental Figure 2:** T_FH_ cells express different subset of cytokine producing cells in CHB patients. (A) Flowcytometric plot indicate the gating strategy used for cytokine producing T_FH_ cells analysis (B,C) HBV-specific and global cytokines including IFN-γ, IL-4, IL-17A and IL-22 producing cells were analyzed in T_FH_ cells after 5 days stimulation with PepMix HBV large envelop protein Ultra (HBs) which has a mix of 216 peptides (15mers with 11 aa overlap) and HBV capsid/core protein which has a pool of 44 peptides, at a concentration of 1μg/ml in the presence of CD49d and CD28 (2μg/ml). Global cytokine producing cells were measured after overnight PMA/ionomycin stimulation. Data analysis was done using flowJo (C) IFN-γ, IL-4, IL-17A and IL-22 cytokines were analyzed in supernatant collected from sorted T_FH_ cells, which were stimulated with PMA/ionomycin for overnight (D) Plasma cytokine level. Flow cytometric cytokine producing cell analysis was conducted in fourteen HC-vacc and CHB in each group. Four HC-vacc and three CHB patients were taken to measure cytokines in sorted T_FH_ cells supernatant, Plasma cytokines were detected in nine CHB and Hc-vacc in each group. Bars indicate median with range or mean with standard deviation. P values were determined either by non-parametric, 2-tailed Mann-Whitney *U* test or unpaired t test. UD: undetected

**Supplemental Figure 3**


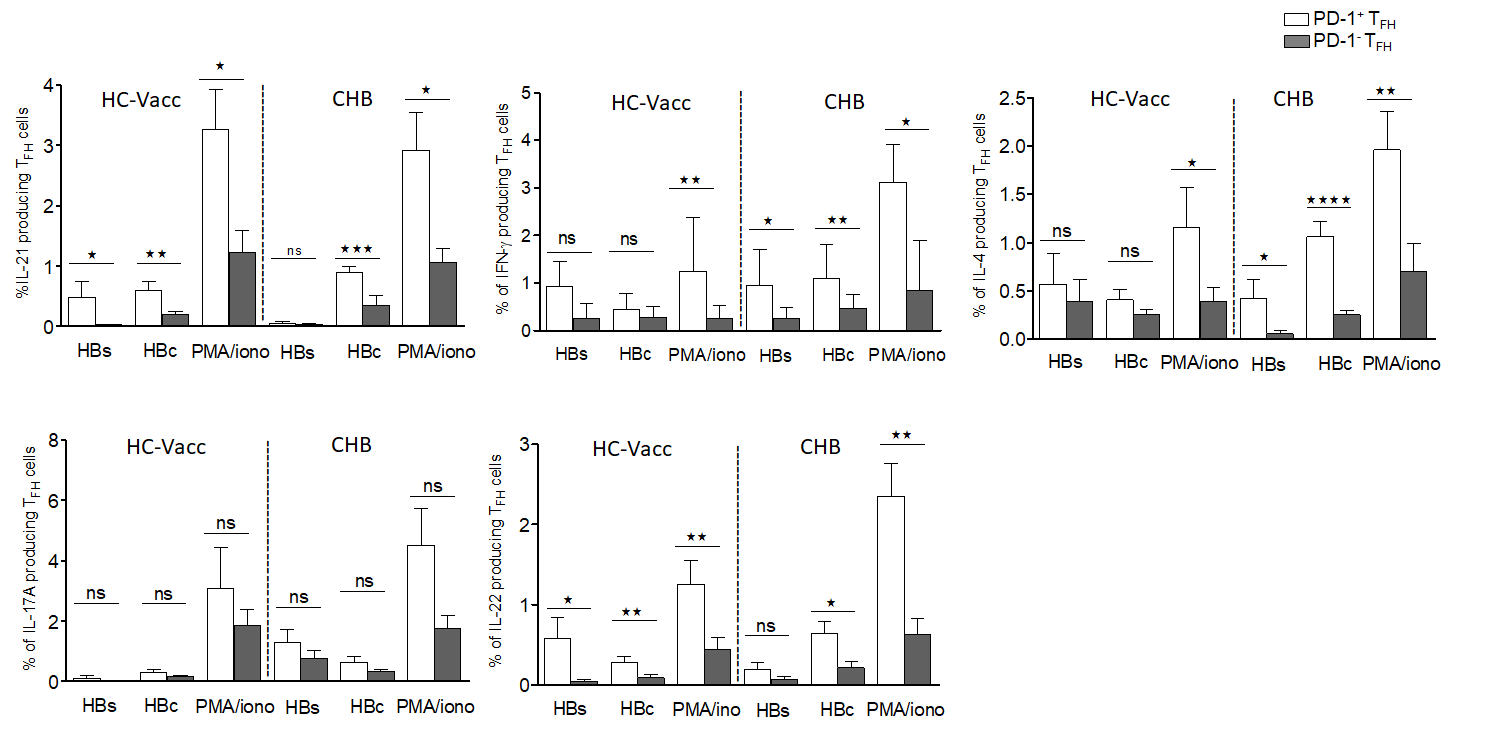


**Supplemental Figure 3.** T_FH_ cell related cytokine producing cells were higher in PD-1^+^ fraction of T_FH_ cells. Peripheral blood mononuclear cells were stimulated with HBs, HBc peptides along with CD28 and CD49d for 5 days. PMA/ionomycin stimulation was performed for overnight. Cytokine producing cells were analyzed in PD-1^+^ and PD-1^-^ fraction of T_FH_ cells. * indicates p<0.05, ** p<0.01, *** p<0.001, **** p<0.0001
